# Pharmacological Effects and Molecular Protective Mechanisms of Astragalus Polysaccharides on Nonalcoholic Fatty Liver Disease

**DOI:** 10.3389/fphar.2022.854674

**Published:** 2022-03-03

**Authors:** Jing Zhang, Quansheng Feng

**Affiliations:** College of Basic Medicine, Chengdu University of Traditional Chinese Medicine, Chengdu, China

**Keywords:** astragalus polysaccharides, metabolic dysfunction-associated fatty liver disease, nonalcoholic fatty liver disease, lipid metabolism, insulin resistance, inflammation, oxidative stress, endoplasmic reticulum stress

## Abstract

Nonalcoholic fatty liver disease (NAFLD) has been renamed metabolic dysfunction-associated fatty liver disease (MAFLD), a condition for which there is now no authorized treatment. The search for new medications to treat MAFLD made from natural substances is gaining traction. The function of anti-oxidant, anti-inflammation, hypoglycaemic, antiviral, hypolipidemic, and immunomodulatory actions of Astragalus polysaccharides (APS), a chemical molecule isolated from Astragalus membranaceus, has become the focus of therapeutic attention. We have a large number of papers on the pharmacological effects of APS on NAFLD that have never been systematically reviewed before. According to our findings, APS may help to slow the progression of non-alcoholic fatty liver disease (NAFL) to non-alcoholic steatohepatitis (NASH). Lipid metabolism, insulin resistance (IR), oxidative stress (OS), endoplasmic reticulum stress (ERS), inflammation, fibrosis, autophagy, and apoptosis are some of the pathogenic pathways involved. SIRT1/PPARα/FGF21, PI3K/AKT/IRS-1, AMPK/ACC, mTOR/4EBP-1/S6K1, GRP78/IRE-1/JNK, AMPK/PGC-1/NRF1, TLR4/MyD88/NF-κB, and TGF-β/Smad pathways were the most common molecular protective mechanisms. All of the information presented in this review suggests that APS is a natural medication with a lot of promise for NAFLD, but more study, bioavailability studies, medicine type and dosage, and clinical proof are needed. This review could be useful for basic research, pharmacological development, and therapeutic applications of APS in the management of MAFLD.

## 1 Introduction

**FIGURE 1:**
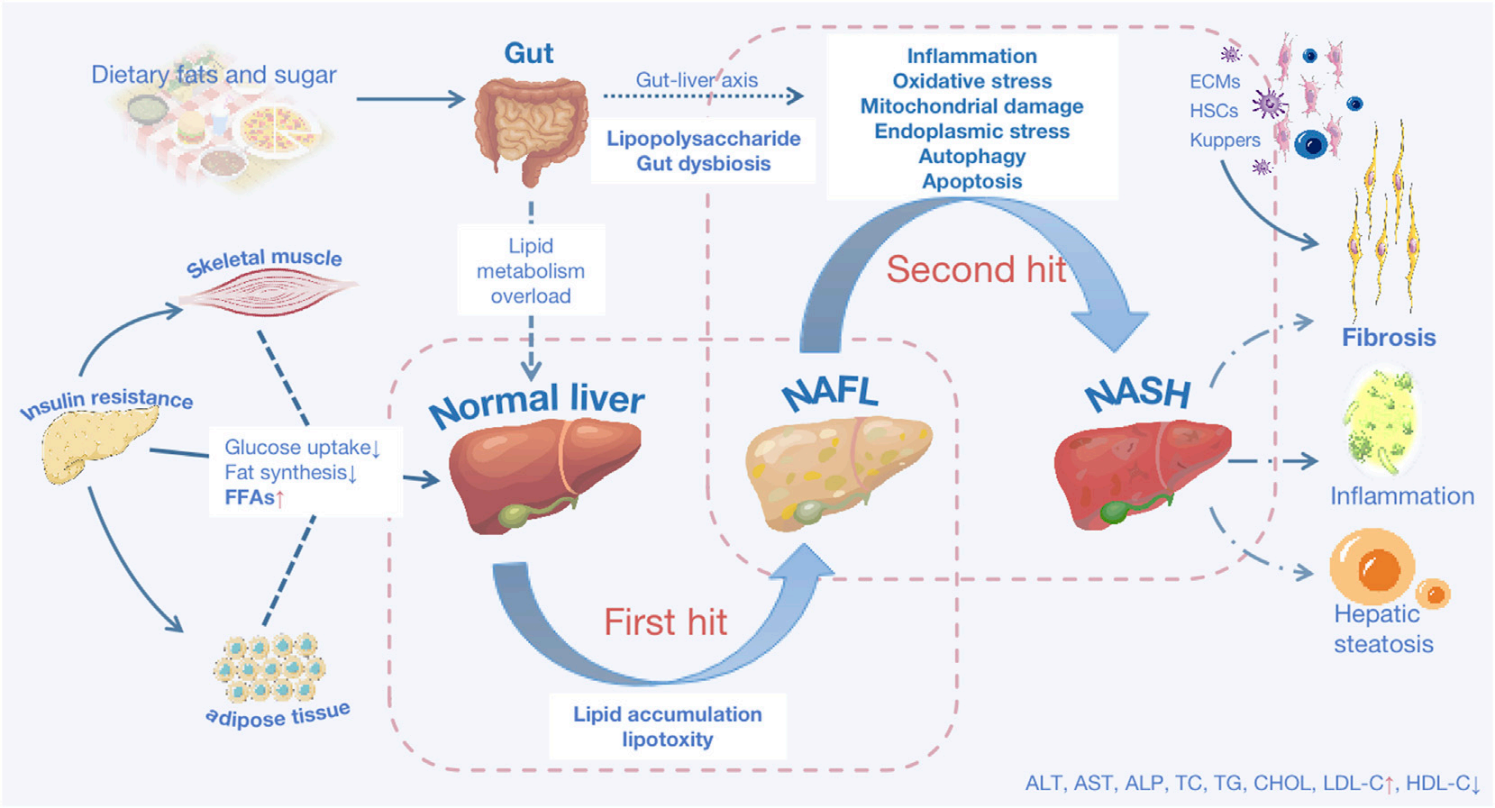
The basic pathological mechanisms of NAFLD. NAFLD is characterized as either non-alcoholic fatty liver disease (NAFL) or non-alcoholic steatohepatitis (NASH) predicated on histological characteristics. Lipid accumulation (LA) is the first hit, and with that lipotoxicity triggered, mainly results from three sources: increased visceral adipose tissue lipolysis, hepatic *de novo* lipid (DNL) production activation, and excessive fat and calorie intake from diets. Insulin resistance, visceral adiposity, and atherogenic are all linked to aberrant LA. Deficient insulin sensitivity in adipose tissue and skeletal muscle, resulting in decreased fat synthesis and glucose uptake, an increase in the quantity of free fatty acids (FFAs) in the blood. FFAs enter the liver as a result of metabolic overload, which is the primary site of fat production. Oxidative stress, endoplasmic reticulum stress, mitochondrial damage, inflammatory reactions, fibrosis, and other factors all contribute to the second hit. Excessive deposition of extracellular matrix (ECM), activation of hepatic stellate cells (HSCs) and kupffer cells contribute to fibrosis in long-term chronic inflammatory reactions. Steatosis causes autophagy and apoptosis in hepatocytes. AST: aspartate transaminase; ALT: alanine aminotransferase; ALP: alkaline phosphatase; TC: total cholesterol; TG: triacylglycerol; CHOL: cholesterol; LDL-C: low-density lipoprotein cholesterol; HDL-C: high-density lipoprotein cholesterol.

In addition to being caused by alcohol and other definitive factors, metabolic dysfunction-associated fatty liver disease, formerly known as nonalcoholic fatty liver disease (NAFLD) (Eslam et al., 2020a; Eslam et al., 2020b), is a clinical-pathological syndrome characterized by steatosis of hepatic parenchymal cells and inflammation in the liver lobes linked to insulin resistance and hereditary predisposition. It is further divided into non-alcoholic fatty liver disease (NAFL) and non-alcoholic steatohepatitis (NASH) based on liver histological changes (Ratziu et al., 2010; Younossi et al., 2018; Eslam et al., 2020b). The related pathogenic process is complex and cascaded-connected, as the “two-hit” theory suggests (Friedman et al., 2018). Fat buildup in the liver is the first essential step in the development of NAFLD. The second hit from NAFL to NASH is triggered by inflammatory reactions, oxidative stress (OS), endoplasmic reticulum (ER) stress, mitochondrial damage, fibrogenesis, and disturbance of the gut flora, among other factors (Pierantonelli and Svegliati-Baroni, 2019) (As shown in Figure 1). NAFLD is the most common chronic liver disease, threatening nearly a quarter of the world’s population (Lonardo et al., 2016), and it will progress to cirrhosis and liver cancer in advanced stages, yet there is still no approved medication to treat it. Therefore, research is urgent and high-profile.

**FIGURE 2:**
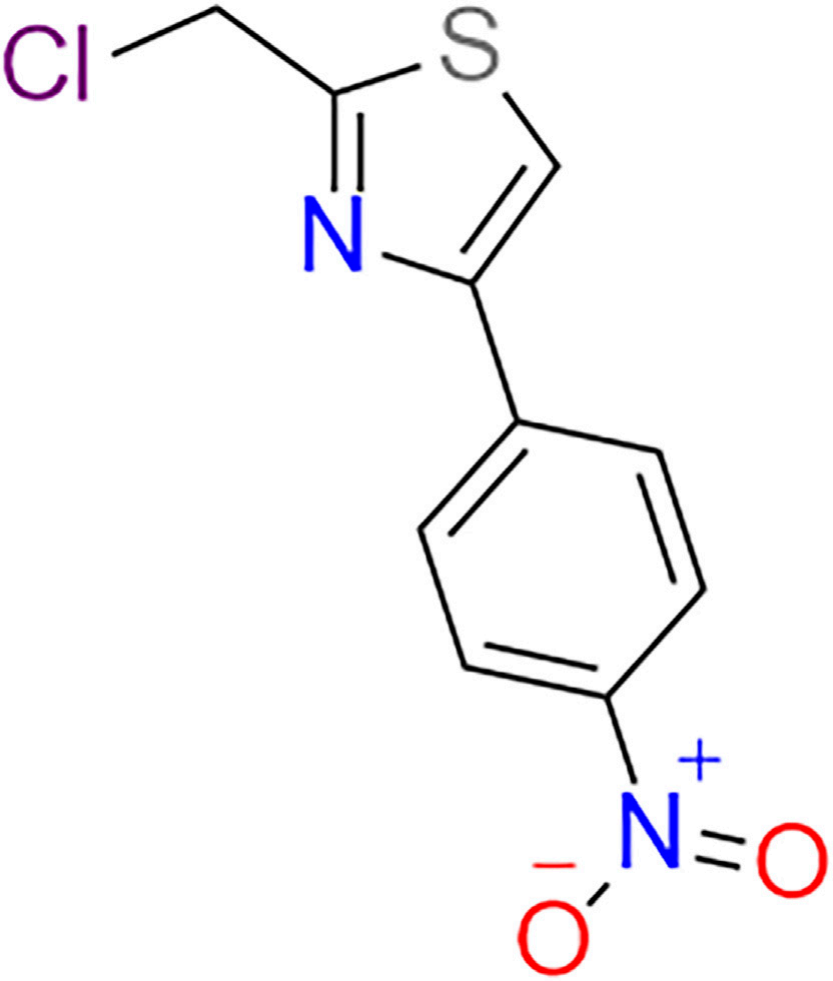
The chemical construction of APS.

Chinese herbal medicine, which is a tremendous treasure trove of human medical development, has indeed demonstrated tangible advantages in the treatment of liver illness. Using contemporary technologies to excavate this treasure trove of potent medicinal components, more and more small-molecule compounds have been discovered from Chinese herbs, such as Flos inulae (Yang et al., 2021), Forsythiaside A (Gong et al., 2021), and Baicalin (Hu et al., 2021). Astragalus membranaceus (AM), also known in China as *Huangqi*, has been used to replenish qi and blood for over two millennia and is suitable for deficiency disorders (Auyeung et al., 2016). Astragalus polysaccharides (2-(Chloromethyl)-4-(4-Nitrophenyl)-1,3-Thiazole, APS) (As shown in Figure 2), a key active ingredient isolated from AM that contains over 200 constituents (Guo et al., 2019), has received a lot of attention over the past 2 decades. A slew of recent pharmacology studies has shown its benefits on blood lipids and glucose management, and it has anti-inflammation, anti-oxidative stress, anti-fibrosis, liver protection, anti-tumor, and immunoregulatory capabilities (Qi et al., 2017; Chen et al., 2020), all of which are linked to the pathogenic mechanism of NAFLD. These NAFLD pathogenic processes are interconnected, promote each other, and share underlying mediators and pathways.

Multi-components, multi-targets, and multi-pathways all seem to be advantages for Chinese medicine. Obtained from experimental evidence on APS and the complex pathophysiology of NAFLD, we assume the potential efficacy of APS for NAFLD. A complete assessment of pharmacological data, particularly on molecular protective mechanisms, is required to stimulate further study and to understand the potential of this medicine; consequently, we wrote this work to encourage further exploration and understanding of its potential.

In this work we intend to provide a complete review of APS’s pharmacological effects and advancements in molecular mechanism works of literature in NAFLD. The flaws in existing development research are also explored, as well as prospective study directions.

## 2 Pharmacological Effects and Molecular Mechanisms of Astragalus Polysaccharides Against Nonalcoholic Fatty Liver Disease

**FIGURE 3:**
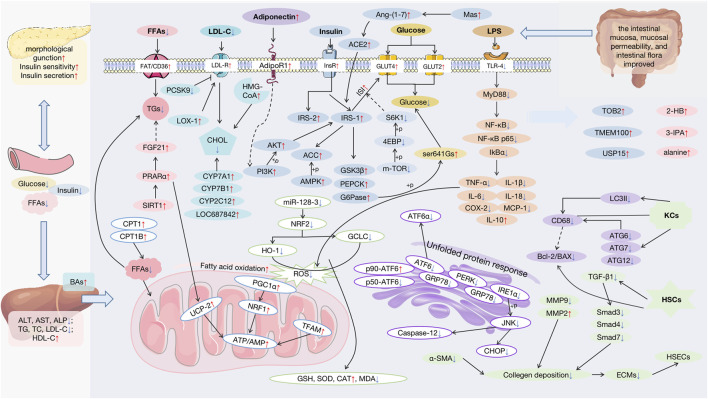
Pharmacological effects and molecular mechanisms of APS against NAFLD. FFAs: free fatty acids; AST: aspartate transaminase; ALT: alanine aminotransferase; ALP: alkaline phosphatase; TG: triglyceride; TC: total cholesterol; LDL-C: low-density lipoprotein cholesterol; HDL-C: high-density lipoprotein cholesterol; BAs: bile acids; FGF21: fibroblast growth factor 21; PPARα: peroxisome proliferator-activated receptor alpha; SIRT1: stimulating the sirtuin 1; CPT1: carnitine palmitoyltransferase 1; LDL-R: low-density lipoprotein receptor; PCSK9: proprotein convertase subtilisin/kexin type; LOX-1: oxidized-LDL receptor-1; CHOL: cholesterol; CYP: cholesterol hydroxylase; HMG-CoA: 3-hydroxy-3-methyl glutaryl coenzyme A reductase; InsR: insulin receptor; Ang-(1–7): angiotensin-(1–7); ACE2: angiotensin-converting enzyme 2; IRS: insulin receptor substrates; ACC: acetyl-CoA carboxylase; AMPK: AMP-activated protein kinase; AKT: protein kinase B; PI3K: the phosphatidylinositol-3 kinase; GSK3β: the glycogen synthase kinase 3beta; PEPCK: the phosphoenolpyruvate carboxyl kinase; G6Pase: the gluconeogenic enzymes glucose 6-phosphatase; S6K1: S6 kinase 1; 4EBP: 4E-binding protein; GRP78: glucose-regulated protein 78; ATF6: transcription factor 6; PERK: protein kinase-like endoplasmic reticulum kinase; IRE1α: inositol-requiring enzyme 1; JNK: the c-Jun N-terminal kinase; CHOP: C/EBP-homologous protein; NRF1: the nuclear factor erythroid 2-like 1; HO-1: heme Oxygenase-1; GCLC: glutamate-cysteine ligase; ROS: reactive oxygen species; PGC1α: the peroxisome proliferator-activated receptor γ coactivator 1; NRF2:; nuclear erythroid-derived 2-related factor 2; UCP-2: uncoupling protein 2; TFAM: the mitochondrial transcription factor A; GSH: glutathione; SOD: superoxide dismutase; CAT: peroxidase catalase; MDA: malondialdehyde; LPS: lipopolysaccharide; TLR4: toll-like receptor 4; MyD88: the adaptor protein myeloid differentiation primary response 88; NF-κB: the nuclear factor-kappa B; IkBα: inhibitory kappa B alpha; TNF-α: tumor necrosis factor-alpha; IL-1β: interleaukin-1β; IL-6: interleaukin-6; IL-18: interleaukin-18; COX-2: cyclooxygenase-2; MCP-1: monocyte chemoattractant protein-1; IL-10: interleaukin-10; LC3II: protein Ⅱ light chain 3; ATG: recombinant autophagy-related protein; KCs:; HSCs: hepatic stellate cells; HSECs: hepatic sinusoidal endothelial cells; TGF-β1: transforming growth factor-β1; ECM: extracellular matrix; TOB2: transducer of ErbB2.2; USP15: the ubiquitin-specific proteases 15; 2-HB: 2-hemoglobin; 3-IPA: 3-indolepropionic acid.

A plethora of research indicates that APS appear to be effective on NAFLD. (As shown in Supplementary Table S1). Serum aspartate transaminase, alanine aminotransferase, and alkaline phosphatase, which indicate liver injury, were all reversed in the study (Hamid et al., 2017a; Hamid et al., 2017b; Sun et al., 2019). Through various mechanisms, APS may exert liver-protecting functions, including improving insulin resistance (IR) and lipid metabolism; inhibiting OS, ER, and mitochondrial injury; providing anti-inflammatory and anti-fibrosis functions; and regulating autophagy and apoptosis, and the main targets modulated by APS are shown in Figure 3.

### 2.1 Lipid Metabolism

The first crucial step in the development of NAFLD is lipids-aberrant accumulation in the liver. Triglycerides (TGs), phospholipids, glycolipids, cholesterol (CHOL), and cholesterol ester are lipids that are part of the cell structure and play a role in cellular homeostasis, cell–cell interaction, and inflammation and immune modulation (Marra and Svegliati-Baroni, 2018). Fatty liver accumulation is caused by a lipid deposition and removal imbalance to toxic lipids species including TGs, free fatty acids (FFAs), lysophosphatidylcholine, ceramides, and free cholesterol (Fch), which results in lipotoxicity and glucotoxicity (Marchesini et al., 2016; Marra and Svegliati-Baroni, 2018; Régnier et al., 2019).

APS could inhibit the synthesis and accumulation of TGs by regulating the transport and breakdown of FFAs (Mao and Ouyang, 2007; Song et al., 2014). FFAs determine the synthesis of TGs, and APS could increase the FA translocator/receptor FAT/CD36 to promote this synthesis (Song et al., 2014). Meanwhile, carnitine palmitoyltransferase 1 (CPT1) and fatty acid oxidation rate-limiting enzyme gene CPT1B increased (Cui et al., 2015b), which helped increase FFA decomposition as a rate-limiting enzyme of long-chain FFAs entering the human mitochondrial inner membrane to speed up the fatty acid oxidation process.

APS may also play a role in the synthesis, catalysis, and metabolism of CHOL. Total cholesterol, Fch, and low-density lipoprotein (LDL) cholesterol levels fell after APS intervention (Cui et al., 2015a; Yan Y. et al., 2020; Wang and Sun, 2021), while high-density lipoprotein (HDL) cholesterol levels increased to move CHOL out (Chen et al., 2018; Cai et al., 2020). The LDL receptor pathway is responsible for the breakdown of LDL, which is a cholesterol-rich lipoprotein. APS could reduce proprotein convertase subtilisin/kexin type 9 (PCSK9) levels via the PPAR-β/γ pathway to increase the expression of low-density lipoprotein receptors (LDL-R) to reduce lipid deposition (Zou, 2016; Sabatine, 2019), and oxidized-LDL receptor-1 (LOX-1) increased to improve lipid disorder (Cui et al., 2015b). It is reported PCSK9 also has a mutually beneficial interaction with LOX-1 and induces the secretion of pro-inflammation cytokines by regulating toll-like receptor 4 (TLR4) expression and NF-κB activation (Ding et al., 2020). It is thought that viscous polysaccharides can lower cholesterol and bile acids (BAs) absorption, which is restricted by APS (Cheng et al., 2011; Cui et al., 2015b; Sun et al., 2019). By inhibiting 3-hydroxy-3-methyl glutaryl-coenzyme A reductase, a rate-limiting enzyme in hepatocyte cholesterol synthesis catalyzing the creation of mevalonic acid, APS may lower CHOL synthesis (Cheng, 2010). To enhance BAs secretion, APS could stimulate the expression of cytochrome P450 (CYP) enzymes such as cholesterol 7-hydroxylase (CYP7A1), cholesterol 7-hydroxylase (CYP7B1), CYP2C12, and LOC687842 (Cheng, 2010; Cheng et al., 2011; Cui et al., 2015b). The alternate pathway to BAs begins with the 27-hydroxylation of cholesterol as the initial step in the liver or extrahepatic tissues, followed by CYP7B1-dependent oxidation in the 7-position (Leoni and Caccia, 2011; Kakiyama et al., 2020). CYP7A1 could transfer cholesterol to HDL particles, which are then returned to the liver for conversion into BAs. CYP2C12 and LOC687842 belong to the family of cytochrome P450 as short hairpin RNA, which participate in the metabolism of the linoleic acid pathway (Elfaki et al., 2018).

By stimulating the sirtuin 1 (SIRT1)/peroxisome proliferator-activated receptor alpha (PPARα)/Fibroblast growth factor 21 (FGF21) pathway, APS could alleviate lipid metabolism disorder, notably, by increasing hepatic glycolipid metabolism, reducing inflammation, and lipid droplet deposition (Gu et al., 2015). PPARα is mainly involved in the catabolism of hepatic lipids and bile acids metabolism, fatty acid uptake, and activation, which affects intracellular fatty acid-binding and mitochondrial fatty acid oxidation (Burri et al., 2010). FGF21 and SIRT1 interact with PPARα to regulate hepatic glycolipid metabolism, OS, and inflammation and may serve as a biological target in NAFLD to improve IR (Ding et al., 2017).

Furthermore, Li et al. discovered the three following distinct metabolites in the APS treatment group and high-fat diet model mice: 3-indole propionic acid, 2-hemoglobin, and alanine, which may ameliorate lipid droplets, but there is no further evidence to support its exact mechanism (Li et al., 2019).

### 2.2 Insulin Resistance

Insulin sensitivity in muscle and adipose tissue declines in the IR state, glucose delivery to these tissues decreases, and FFAs are liberated into the systemic circulation. To compensate for high blood glucose (BG) levels, pancreatic beta cells release more insulin, resulting in hyperinsulinemia. Despite being primarily insulin-sensitive and being exposed to high concentrations of BG, TGs, FFAs, and insulin, the liver enters a hyper-anabolic state when it continues to generate and store lipids, all of which is improved by APS (Gu et al., 2015; He et al., 2016). APS may help injured islets regain their morphological function and maintain a balance of normal insulin output and compensatory insulin secretion (Gu et al., 2015; Hong et al., 2020; Liu et al., 2020).

APS may increase ISI by stimulating the phosphorylation of insulin receptor substrates (IRS) and aiding the transit of glucose transporters (GLUTs) from the nucleus to the cell membrane, promoting glucose transfer and boosting insulin utilization (Zou et al., 2009; Sun, 2012; Ye et al., 2014; Sun et al., 2019). Insulin insufficiency is characterized by a lack of receptors and a reduction in GLUTs. APS may phosphorylate IRS-1 by the phosphatidylinositol-3 kinase (PI3K)/protein kinase B (PKB, also known as Akt) signal pathway to promote the activation of the AMPK/acetyl-CoA carboxylase (ACC) pathway and its downstream targets, glycogen synthase kinase 3beta (GSK3β), phosphoenolpyruvate carboxyl kinase (PEPCK), and gluconeogenic enzymes glucose 6-phosphatase (G6Pase) phosphorylation, to improve glucose metabolism (Sun et al., 2019). The metabolic function of insulin is mainly achieved through the PI3K signal pathway, which binds to the insulin receptor to phosphorylate IRS, leading to Akt phosphorylation; it exerts the biological effects of insulin to promote glucose uptake and glucose homeostasis in the liver. PEPCK and G6Pase regulate gluconeogenesis (Liu Q. et al., 2017), GSK3β could inhibit glycogen synthesis, and the synthesis also reduced results from ser641GS phosphorylation activated by APS (Zou et al., 2007). AMPK is the main energy sensor that regulates the dynamic balance of ATP/AMP levels up-regulated and phosphorylated by APS (Zou et al., 2009; Zou, 2010; Sun et al., 2019). Lipoglycometabolism, insulin sensitivity, and plasma glucose regulation are all dependent on it (Smith et al., 2016). APS might boost IRS-1 and Akt phosphorylation, triggering the PI3k signal transduction pathway to improve ISI by activating the angiotensin-converting enzyme 2-Ang-(1–7)-Mas axis and inhibiting the mTOR/4E-binding protein 1 (4EBP-1)/S6 kinase 1 (S6K1) signaling pathway (Simanshu et al., 2017; Santos et al., 2018; Ma et al., 2019; Sun et al., 2019); excessive intake of energy or insulin resistance causes overexpression of mTOR, which activates 4EBP-1 and S6K1 phosphorylation. APS promote glucose synthesis by activating GLUT4 and GLUT2 translocation to improve ISI. GLUT4 and GLUT2 are sorted and retained intracellularly, and they dynamically redistribute to the plasma membrane by insulin-regulated vesicular traffic (Thorens, 2015; Jaldin-Fincati et al., 2017). APS could also promote adiponectin secretion to activate the downstream PI3K/Akt/IRS-1 pathway by increasing its receptor AdipoR1 levels (Sun et al., 2019). Changes in morbid adipose tissue in endocrine characteristics could decrease adiponectin secretion, which is an insulin enhancer (Hamid et al., 2017a).

Metabolomics revealed that APS could strengthen ISI by upregulating the mi-RNA transducer of ErbB2.2, TMEM100, and the ubiquitin-specific proteases (USP15) (Liu et al., 2020). There is no direct evidence on IR about the mi-RNAs, but they all affect inflammation, which may influence ISI indirectly (Pan et al., 2019; Jiang et al., 2020; Das et al., 2021). Studies have shown that both the let-7 family and miR-103 can modulate ISI in the liver, and both are the target genes of TMEM100 and USP15, which may explain why APS improve IR (Chakraborty et al., 2014; Liu et al., 2020). However, more experimental validation is required.

### 2.3 Oxidative Stress, Endoplasmic Reticulum, and Mitochondrial Injury

In NASH patients, studies revealed a positive relationship between OS levels and IR severity, fat degeneration, inflammatory response, and fibrosis (Tariq et al., 2014). ROS are produced primarily by mitochondrial failure, ER stress, particulates, and peroxidase oxidation (Scherz-Shouval and Elazar, 2007; Forrester et al., 2018; Das et al., 2021), all of which are reduced by APS to alleviate liver injury (Wang, 2016). The increased input of fatty acids and oxidation in mitochondria, on the other hand, is a primary source of ROS in NAFLD (Forrester et al., 2018).

#### 2.3.1 Oxidative Stress

OS is a balance between free oxygen radical generation and antioxidant protection (Forrester et al., 2018). Studies have shown APS could regulate the levels of the final products of membrane lipid peroxidation malondialdehyde (MDA), antioxidant metal enzyme superoxide dismutase (SOD), and redox agent glutathione (GSH), which are OS damage indicators and clear the content of hydroxyl free radicals and superoxide anions (Tong et al., 2006; Wang et al., 2015; Wang X. et al., 2016; Yang, 2017; Yuan et al., 2018), both of which are common free oxygen radicals with lipotoxicity (Mota et al., 2016).

APS could activate the nuclear erythroid-derived 2-related factor 2 (NRF2) pathway and its downstream protein glutamate-cysteine ligase and heme oxygenase-1 (HO-1) to anticipate anti-oxidative stress, reversing the marker enzyme of peroxidase catalase, MDA, SOD, and GSH-Px after suffering OS damage (Yan Y. et al., 2020; Qu et al., 2020). NRF2 is a determinant regulating the transcription of antioxidant enzyme lines, which could regulate the expression of more than 100 genes (Wang et al., 2019). Yuan et al. showed that APS regulate the NRF2 pathway, which might be dependent on inhibiting miR-128-3 that can regulate the level of antioxidant enzymes and play a therapeutic role in hyperlipidemia by *in vivo* and *in vitro* experiments (Yuan et al., 2018).

#### 2.3.2 Endoplasmic Reticulum Stress

ER is the main cellular compartment involved in secretory and transmembrane protein productive folding, lipid biogenesis, and calcium homeostasis (Lebeaupin et al., 2018). With the occurrence of abnormal glycolipid metabolism, cells activate unfolded proteins in response to misfolded and unfolded protein aggregation and calcium balance in the ER, and misfolded proteins induce ROS generation while OS disturbs the ER redox state, thereby breaking the correct disulfide bond formation and proper protein foldin (Wang J. et al., 2016).

APS improve ER and the resulting autophagy by inhibiting the ATF6 passage and glucose-regulated protein 78 (GRP78)-related pathways [including IRE-1α, the c-Jun N-terminal kinase (JNK), and PERK], reducing the apoptosis factors expression of C/EBP-homologous protein and caspase-12 (Wang et al., 2009; Hu et al., 2010; Wei et al., 2018). Under ER stress, partner GRP78 binds to unfolded protein, releasing the following three transmembrane receptors: inositol-requiring enzyme 1 (IRE-1α), protein kinase-like endoplasmic reticulum kinase (PERK), and activating transcription factor 6alpha, allowing their signaling pathways to be activated. The dephosphorylation of PERK and IRE1 could be initiated by APS and suppress p50-ATF6 and activate p90-ATF6 to improve ER and recover glucose homeostasis (Wang et al., 2007; Wang et al., 2009).

#### 2.3.3 Mitochondrial Injury

Increased fatty acid oxidation and lipotoxicity in NASH are principal drivers of mitochondrial deterioration (Schuppan et al., 2018). The liver adapts to the influx of excess fatty acids caused by liver lipid deposition, increasing mitochondrial β oxidation and the number of mitochondria. It is not only a direct reaction to fatty acids but also an activation of lipid cytokines caused by IR and increasing PPAR-α activation levels, which is inhibited by APS as shown earlier. NAFLD patients show a rise in pro-inflammation cytokine TNF-α levels. Remarkably, APS may reduce TNF-α levels while inhibiting the expression of PPAR-α (Chen et al., 2017).

By stimulating the expression of uncoupling protein 2, APS could improve liver energy metabolism disorders and limit the production of ROS, up-regulating ATP enzyme activity (Gu et al., 2015; Kan, 2018). UCPs are proteins that exist on the inner membrane of mitochondria, limiting the production of ROS by mitochondria, and protecting cells from oxidative damage caused by mitochondrial respiration (Toda and Diano, 2014). High levels of UCPs may lead to high levels of protein leakage and uncoupling of substrate oxidation from ADP phosphorylation, thereby limiting ATP synthesis and energy loss (Demine et al., 2019).

Besides, APS may promote mitochondrial biogenesis through the AMPK-mediated peroxisome proliferator-activated receptor γ coactivator 1 (PGC-1α)/nuclear factor erythroid 2-like 1 (NRF1) signaling pathway, up-regulating the mitochondrial transcription factor A level to improve mitochondrial oxidative phosphorylation levels and boost mitochondrial function and mitochondrial DNA replication (Cheng et al., 2018; Kan, 2018; Kang et al., 2018).

### 2.4 Inflammation, Fibrosis, Autophagy, and Apoptosis

Long-term repetitive inflammation irritation is an accomplice to the progression of NAFLD, further leading to sustained hepatic fibrogenesis and, ultimately, cirrhosis (Koyama and Brenner, 2017). Apoptotic hepatocytes activate quiescent hepatic stellate cells (HSCs) and Kupffer cells (KCs) that in turn promote inflammation and fibrosis (Brenner et al., 2013). Inflammation, fibrosis, autophagy, and apoptosis as inter-related pathogenesis deteriorate each other in the progression of NAFLD.

#### 2.4.1 Inflammation

Inflammatory damage as a result of the imbalance between pro-inflammatory cytokines and anti-inflammation is triggered by various endogenous or exogenous factors in adipose tissue or the gut, such as lipotoxicity, cells apoptosis, innate immune responses, OS, mitochondrial dysfunction, and ER (Schuster et al., 2018). APS could down-regulate inflammation-related pathway proteins and cytokines *in vitro* and *in vivo*, such as a series of pro-inflammatory cytokines including tumor necrosis factor-alpha (TNF-α), interleukin-1beta, interleukin-6, interleukin-18, cyclooxygenase-2, and monocyte chemoattractant protein-1 (MCP-1/CCL2), which up-regulate anti-inflammation cytokine interleukin-10 (Cui et al., 2016; Sun et al., 2019; Cai et al., 2020).

By enhancing the intestinal mucosa, mucosal permeability, and intestinal flora, APS protect against endotoxin generated by intestinal bacteria that enter via the portal circulation to activate toll-like receptor-4 signaling in Kupffer cells (Mollazadeh et al., 2019; Wang and Sun, 2021). The entry of lipopolysaccharide into the liver via the damaged intestinal mucosal barrier stimulates the release of pro-inflammatory cytokines, and the downstream signaling pathways activation of the adaptor protein myeloid differentiation primary response 88 (MyD88) and nuclear factor-B (NF-κB) have been demonstrated to be inhibited in the APS treatment group to alleviate liver steatosis (Zhang et al., 2012); the associated proteins NF-κB, NF-κB p65, and inhibitory kappa B alpha were lowered, as were the regulatory proteins governing innate and adaptive immune responses (Wang et al., 2013; Hamid et al., 2017b; Freitas and Fraga, 2018).

#### 2.4.2 Fibrosis

Hepatic fibrosis develops as a consequence of a chronic, recurrent wound healing process (Schuppan et al., 2018). Cascade responses (such as OS, necrosis, and apoptosis) induced by liver cell damage are intimately linked to inflammation and can serve as a risk indicator for the development of hepatic fibrosis (Koyama and Brenner, 2017; Schuppan et al., 2018). APS inhibit fibrosis formation by inhibiting oxidative stress, apoptosis, and inflammation; enhancing immunity; and preserving the shape and function of sinusoidal and hepatic cells, as well as reducing extracellular matrix (ECM) deposition (Niu et al., 2012; Xu et al., 2012; Zhang, 2012; Li, 2013; Huang et al., 2015).

Hepatic fibrosis is characterized by the deposition of the ECM, a rise in myofibroblasts, and hyperplasia of fibrous tissue. Additionally, pseudolobules occur, which are reversed by APS (Zhang et al., 2009; Qin, 2012; Huang et al., 2015; Zhang et al., 2015). Collagen, proteoglycan, laminin, fibronectin, and matrix cell proteins are all deposited as ECMs. APS may decrease collagen deposition by inhibiting the activation of Kupffer cells and the hyperplasia of HSCs (Qin, 2012; Huang et al., 2015). The activation indicators of HSCs transforming growth factor-beta1 (TNF-β1), and alpha-smooth muscle actin were reduced in the presence of APS (Huang et al., 2015; Zhang et al., 2015; Hamid et al., 2017a; Hamid et al., 2017b). Likewise, APS may decrease MMP9 expression and boost MMP2 to maintain a balance between matrix metalloproteinases (MMPs) and their inhibitors, hence promoting ECM deposition. MMP2 and MMP9 are gelatinases that could also cleave type IV collagen and degrade type V, VII, and X collagen, fibronectin, and elastin (Pittayapruek et al., 2016). ECM deposition in the Disse space results in the establishment of endodermis and the loss of fenestration in hepatic sinusoidal endothelial cells (HSECs). APS may increase defenestration of HSECs, sinusoidal capillaries, and liver cells by increasing Young’s modulus, the fenestration area, and several SECs (Li, 2013; Yan F. et al., 2020), as well as the hepatic circulation flow rate and perfusion (Tian and Xu, 2008).

APS may act as a regulator of the TGF-β/small mother against the decapentaplegic (Smad) pathway, thereby preserving the basement membrane-like intercellular material seen in normal liver tissue and preventing the development of scar tissue (Huang et al., 2015; Hu et al., 2018). TGF-1, Smad3, Smad4, and Smad7 expressions were all suppressed while Smad7 increased CCL4-induced fibrosis in rats (Huang et al., 2015).

It has been reported that astragalus has the function of immunity enhancement (Liu CH. et al., 2017; Qi et al., 2017; Liu and Lv, 2020). Additionally, APS may ameliorate liver damage by boosting immunity, which results in a rise in serum total protein, albumin, and albumin/globulin levels, while decreasing globulin levels (Zou et al., 2002; Niu et al., 2012; Xu et al., 2012).

#### 2.4.3 Autophagy and Apoptosis

Autophagy and apoptosis research in the context of improving NAFLD includes lipid removal and anti-inflammatory and anti-oxidative stress benefits. Hamid et al. observed that APS stimulate KCs by decreasing the amounts of recombinant autophagy-related proteins (ATGs), family members (ATG7, ATG12, and ATG6), and protein II light chain 3 (LC3II), resulting in a decrease in CD68-positive KCs (Hamid et al., 2017b). According to previous studies, ATG7 and LC3II recruited by PI3K further clear hepatocellular lipid droplets, and ATG7 may also control PERK, linking autophagy with ER (Martinez-Lopez and Singh, 2015; Zheng et al., 2019). As shown by the decreased expression of Bcl-2/BAX apoptotic genes, APS may also promote apoptosis in activated HSCs in hepatic fibrosis (Hamid et al., 2017a). The Bcl-2 family of proteins controls and regulates the intrinsic or mitochondrial apoptotic pathway (Peña-Blanco and García-Sáez, 2018). The Bcl-2 protein family regulates and controls the intrinsic or mitochondrial apoptotic process (Mukhopadhyay et al., 2014).

## 3 Conclusion and Future Perspectives

As phytotherapy, APS are appropriate for long-term therapeutic options in patients with chronic illness owing to relatively small toxic side effects, which has been the most difficult issue in the study of NAFLD treatments, resulting in a large number of medicines being abandoned in subsequent clinical trials. Increasing data indicate that APS are useful for NAFLD. It may enhance lipid metabolism by reducing the buildup of lipids such as TGs, FFAs, and CHOL (in particular, the production and secretion of CHOL and Bas). FAT/CD36, CPT1B, PCSK9, LDL-R, LOX-1, CYP enzymes, and the SIRT1/PPARα/FGF21 pathway are the major molecular processes and pathways involved. They have the potential to reverse IR by increasing ISI, restoring islet cell shape and function, controlling insulin secretion, and stimulating glucose absorption, all of which are necessary for proper lipogenesis and glycogen production. APS regulate the PI3K/Akt pathway, phosphorylating IRS-1 and activating the AMPK/ACC pathway to ameliorate inadequate ISI, and down-regulates the mTOR/4EBP-1/S6K1 pathway and the addition of adiponectin. Additionally, the therapeutic impact of APS on NAFLD may be related to its capacity to ameliorate OS, ERS, and mitochondrial damage through regulation of the NRF2/HO-1, GRP78/IRE-1/JNK, and AMPK/PGC-1/NRF1 pathways, as seen in Figure 3. APS inhibit inflammation and fibrosis and regulates autophagy and apoptosis in HSCs and Kupffer cells via the TLR4/MyD88/NF-κB pathway and gut microbiota; ECMs are diminished and proliferation of HSCs and Kupffer cells is inhibited *via* the TGF-β/Smad pathway; HSEC defenestration and immunity are improved; and autophagy and apoptosis occurs in HSCs. Among them are several small-molecule compounds that have been screened using metabolomics, but their findings have not been confirmed in subsequent research, and many of the current animal and cell experiments have remained in the laboratory. Preliminary verification of pathological improvement needs more systematic and in-depth study; further verification of its mechanism of action requires more systematic and in-depth research. At the moment, no clinical data exist to support the therapeutic efficacy of APS in NAFLD. As a consequence of the progress of so many experiment outcomes, further clinical studies will be required in the future to validate the data.

However, because of the intricacy of its structure and composition, it is difficult to correctly regulate the quality of its compounds, and we must make further efforts toward this end (Zeng et al., 2019). APS have been extensively investigated as an immunostimulant and anti-aging, anti-diabetic, anti-tumor, and antiviral agent (Zheng et al., 2020). Owing to the origin of the raw materials, the medicinal components, the growth years of the medicinal materials, and the extraction technique, the purity, composition (e.g., polysaccharide content), and chemical structure of APS will vary (Li et al., 2015). Huang et al. identified polysaccharides as one of the most essential components of Astragalus (Huang et al., 1982). The major components of APS are heteropolysaccharide, neutral polysaccharide, dextran, and acidic polysaccharide, with heteropolysaccharide being the most abundant (Zheng et al., 2020).

As a macromolecular molecule, APS are insoluble in water, have a low oral absorption rate and a low bioavailability, and are quickly affected by stomach acid and other variables, resulting in a highly limited therapeutic effectiveness. In recent years, in conjunction with emerging technologies, a plethora of new preparations aimed at increasing the drug’s absorption rate and efficacy have been developed, such as the establishment of APS liposomes and the preparation of APS microcapsules to extend the drug’s storage time and enhance its slow-release effect and boost immunological function (Fan et al., 2011). Additionally, APS pellets may be manufactured for use as a colon-targeted formulation, allowing the medicine to bypass the intestinal mucosal barrier and reach the liver (Qin et al., 2016; Lai et al., 2017). Chitosan is used to synthesize APS nanoparticles, which boost the herb’s therapeutic efficacy in the treatment of blood disorders (Lai et al., 2017). Additionally, colonized and fermented APS are often employed in anti-liver fibrosis studies (Zhou et al., 2017). In clinical settings, APS injection is used the most. It has been extensively used to increase immune function and aid in the treatment of asthma and hypertension in patients after cancer chemotherapy (Wang et al., 2021). As previously stated, it aims to address the issue of APS’s limited oral availability. That said, as we can see, various preparations alter the functional targeting of APS. Is there a better way to handle APS for the treatment of NAFLD? The gut-liver axis is critical in NAFLD, and APS have also been shown to promote intestinal flora. Not only may APS be used as a pharmaceutical, but it can also be utilized as a dietary supplement for metabolic illness nutritional treatment.

Modern experimental technology has made tremendous strides in recent years. High-throughput techniques such as metabolomics, transcriptomics, epigenetics, and gut microbiota analysis may aid in the exploration of drug action mechanisms. APS demand a more thorough examination of the exploration of NAFLD therapeutic mechanisms by those technologies.
