# Supplementary material for: Pharmacological Effects and Molecular Protective Mechanisms of Astragalus Polysaccharides on Nonalcoholic Fatty Liver Disease
Source: Front Pharmacol. 2022 Mar 3;13:854674. doi: 10.3389/fphar.2022.854674 (PMC8929346; doi:10.3389/fphar.2022.854674)
Supplement: Supplementary file 1 [file Table1.doc]

**Table 1. Protective effect of APS on NAFLD**

| **Model** | **Animal species** | **Dosage** | **Activity/mechanism(s) of action** | **application** | **Reference** |
| --- | --- | --- | --- | --- | --- |
| High-fat diets, 30%(w/v) fructose | ICR mice | 400mg/kg | BW, liver index reduced, liver steatosis, intestinal mucosa permeability, ALT, AST, ALP, TG, TC, GLU, TLR4, MyD88↓, LDL, HDL↑ | in vivo | (Wang et al., 2021) |
| 80mg/kg STZ, i.p. | Wistar rats | 0.2,0.4,0.8g/kg | TG, LDL-C, TNF-α, IL-1β, IL-18↓, HDL-C, IL-10↑ | in vivo | (Cai et al., 2020) |
| High-fat diets, 25mg/kg STZ,i.p. | Wistar rats | 200,400mg/kg | Hepatocellular morphology improved, hyperplasia of bile canaliculus, lipid droplets and the vacuoles in hepatocyte, collegen deposition decreased, hepatic glycogen, MDA↓, SOD, Nrf2, p-Nrf2, HO-1↑ | in vivo | (Qu et al., 2020) |
| High-fat diets, 25mg/kg STZ,i.p. | KM mice | 10ml/kg | Pancreatic bubble cell edema improved, serum insulin, Tob2, Tmem100, Usp15↑ | in vivo | (Liu et al., 2020) |
| High-fat diets | SD rats | 400mg/kg | Liver index decreased, liver steatosis, inflammation and lymphocytic infiltration reduced, ALT, AST, TC,TG, MDA↓, Ang1-7, SOD, ACE2, Mas, IRS-1↑ | in vivo | (Ma et al., 2019) |
| High-fat diets | C57BL / 6J mice | 2%,4%,8%(add into high-fat diets) | Weight gain, liver index , lipid droplets and the vacuoles in hepatocyte deduced, 3-IPA, 2-HB, β-alanine↑ | in vivo | (Li et al., 2019a) |
| High-fat diets,70mg/kg STZ, i.p. | KM mice | 300g/L | Weight gain, hepatocellular arrangement improved, dropsy, steatosis, mitochondria swollen, the degree of hepatic sinusoid dilation, hepatic glycogen decreased, GLU↓ | in vivo | (Li et al., 2019b) |
| High-fat diets,70mg/kg STZ, i.p. | KM mice | 1500mg/kg | Weight gain, Pancreatic bubble cell edema, Islet morphology, mitochondrial swelling and endoplasmic reticulum injury improved, swollen, steatosis of hepatocytes duduced, GLU↓, PINS↑ | in vivo | (Li et al., 2019c) |
| High-fat diets | SD rats | 25mg/kg | IL-6, hsCRP↓, SOD,GSH↑ | in vivo | (Tie et al., 2019) |
| 1mg/kg 40% CCL4, .i.p | SD rats | 0.2,0.4,0.8g/kg | PCIII, IVC, LN, HA↓ | in vivo | (Sun et al., 2019) |
| High-fat diets | SD rats | 700mg/kg | inflammation infiltration of pancreatic islet decreased, shape of islet improved, Pancreatic β cells and granule increased, TC, TG, LDL-C, AMS↓, HDL-C↑ | in vivo | (Chen et al., 2018) |
|  | BRL-3A | 100μg/ml | BRL-3A cells proliferation promoted, Ca2+-Mg2+-ATP,Na+-K+-ATP, T-ATP enzyme activity increased, ATP, ADP, total adenosine pool and horon, number of mitochondria and mtRNA copy, PGC-1α, NRF-1, TFAM, ATPaseβsubunit, AMPK, p-AMPK↑, AMP↓ | in vitro | (Kan et al., 2018) |
| High-fat diets | SD rats | 700mg/kg | Weight gain and liver index decreased, liver steatosis deduced, TC, TG, LDL-C , MDA, T-SOD, AST, ALT, GLU↓, HDL-C↑, | in vivo | (Yuan et al., 2018a) |
| High-fat diets | SD rats；BRL，HepG2 cells | 700mg/kg;150μg/ml | Liver index and steatosis, inflammation infiltration of pancreatic islet decreased, islet swollen improved and granule increased, GLU, AMS, ALT, AST, TC, TG, MDA, miR-128-3p↓, T-SOD, NRF2↑ | in vitro, vivo | (Yuan et al., 2018b) |
| 5,50 μmol/L H_2_O_2；_10ml/kg 0.1% CCL4, i.p. | LO2,HepG2；C57B/L mice | 0.2,0.4,0.6,0.8,1mg/ml；300mg/kg | Liver index deduced, MDA↓, DPPH and OH free radical scavenging rate, O^2^ superoxide ion free radical scavenging rate, CAT, SOD, GSH-Px↑ | in vitro, vivo | (Yang et al., 2017) |
|  | HepG2，THP-1 cells | 600,400μg/ml | PCSK9↓, LDLR↑ | in vitro | (Zou et al., 2016) |
| 200mg/kg STZ, i.p. | Wistar rats | 400mg/kg | Lipid droplets and liver glycogen, mitochondria and endoplasmic reticulum structure damage deduced, AST, ALT, MDA↓, SOD↑ | in vivo | (Wang et al., 2016a) |
| High-fat diets, 40mg/kg STZ,i.p. | Wistar rats | 400mg/kg | Swollen, lipid droplets and the vacuoles of hepatocytes, mitochondria damage improved, GLU, TC,TG, AST, ALT, MDA↓ | in vivo | (Wang et al., 2016b) |
| High-fat diets | SD rats | 100,200,400mg/kg | Liver steatosis improved, TC, TG, LDL-C↓, HDL-C↑ | in vivo | (Tang et al., 2016) |
| High-fat diets | C57BL / 6J mice | 2%、4%、8%(add into high-fat diets) | Liver steatosis, intestinal flora structure improved, TC, TG, HOMA-IR↓ | in vivo | (He et al., 2016) |
| 3ml/kg 60% CCL4, s.c. | SD rats | 0.2,0.4,0.8g/kg | Liver fibrosis improved, AST, ALT, TGF-β1, Smad3，Smad4↓, Smad7↑ | in vivo | (Huang et al., 2015) |
| High-fat diets | KKay mice，C57BL/6J mice | 700mg/kg | GLU, MDA↓, SOD↑ | in vivo | (Wang et al., 2015) |
| 3ml/kg 60% CCL4, s.c. | SD rats | 0.2,0.4,0.8g/kg | Hyperplasia of fibrosis decreased, PCIII, CIV, LN, HA, Hyp, Col-I, α-SMA↓ | in vivo | (Zhang et al., 2015) |
| High-fat and low protein diets, Weight-bearing swimming | Wistar rats | 1.41g/kg | Swollen, lipid droplets and the vacuoles of hepatocytes improved, CYP1A1↓, OLRL, CPT1B, CYP2C12, LOC687842↑ | in vivo | (Cui et al., 2015a) |
| High-fat and low protein diets, Weight-bearing swimming | Wistar rats | 1.41g/kg | ALT, AST, ALP, TG, CHOL, LDL-C↓, HDL-C↑ | in vivo | (Cui et al., 2015b) |
| High-fat diets, 35mg/kg STZ, i.v. | Wistar rats | 400mg/kg | BW deduced, FFA, GLU↓, p-AMPK, FAT/CD36, CPT1↑ | in vivo | (Song et al., 2014) |
| 1mg/L LPS | liver cells iosolated form SD rats | 25,50,100mg/L | Hepatocytes survival rate increased, AST, ALT, TNF-α, IL-6, IkBα, p65↓ | in vitro | (Wang et al., 2013) |
|  | SECs isolated from wistar rats | 12.5,25,50,100,200μg/ml | SECs Young's modulus, fenestration area and number increased, sinusoidal capillaries improved, NO↑ | in vitro | (Li et al., 2013) |
| 2ml/kg 50% CCL4,i.p. | SD rats | 50,100,200 mg/kg | BW increased, liver cells damage, degeneration, collagen fibers, fatty vacuoles deduced, ALT, AST, ALP, PⅢNP, CⅣ, LN, HA, Hyp, MDA↓, T-AOC, GST↑ | in vivo | (Qin et al., 2012) |
| High-fat diets, 25mg/kg STZ, i.p. | SD rats | 400mg/kg | FBG, TC,TG, Fins, VF/W↓, ISI, AMPKα, p-AMPKα↑ | in vivo | (Wang et al., 2012) |
| 1mg/kg CCL4, s.c. | KM mice | 500,1500mg/kg | liver tissue damage, inflammation infiltration improved, ALT↓, TP↑ | in vivo | (Niu et al., 2012) |
| bile duct ligation | SD rats | 700mg/kg | Ascites, fibrosis, spleen weight, degenerration of hepatocytes improved, AST, ALP, TBil, Hyp, TGF-β1↓, Alb↑ | in vivo | (Xu et al., 2012) |
|  | SECs isolated from wistar rats | 50μg/ml | SECs Young's modulus increased | in vitro | (Zhang et al., 2012) |
| High-fat diets, 25mg/kg STZ,i.p. | SD rats | 400mg/kg | Liver steatosis deduced, FBG, TG, TC, Fins↓, ISI, InsR, IRS-2↑ | in vivo | (Sun et al., 2012) |
| High-fat diets, 25mg/kg STZ, i.p. | SD rats，C2C12 cells | 700mg/kg,200μg/ml | GLU, liver gycogen, FBG, RBG, OGTT, HOMA-IR↓, ISI, BAX, p-AMPKα, P-ACC↑ | in vivo | (Zou et al., 2010) |
| High-fat diets, 25mg/kg STZ, i.p. | SD rats | 700mg/kg | BW, BG, HOMA-IR, OGTT↓, CHOP↓ | in vivo | (Hu et al., 2010) |
| High-fat diets | Wistar rats | 0.25g/kg | Vacuolar degeneration of hepatocytes improved, TC, TG, LDL-C, HMG-CoA↓, HDL-C, bile acid and cholesterol excretion, CYP7B1↑ | in vivo | (Cheng et al., 2010) |
| High-fat diets, 25mg/kg STZ,i.p. | SD rats | 700mg/kg | FBG, RBG, OGTT, HOMA-IR, IRE1, p50-ATF6↓, p90-ATF6↑ | in vivo | (Wang et al., 2009) |
| 2ml/kg 40% CCL4, s.c., 10% ethnol drink | SD rats | 200mg/kg | Lipid droplets in hepatocytes and hyperplasia of HSCs, fibrosis, kupffer cells deduced, Hyp↓ | in vivo | (Zhang et al., 2009) |
| High-fat diets | C57BL / 6J, KKAy mice | 700mg/kg | Swollen, steatosis of hepatocytes improved, GLU, HOMA-IR↓, hepatic glycogen, p-ser641GS↑ | in vivo | (Zou et al., 2007) |
| High-fat diets, 25mg/kg STZ, i.v. | SD rats | 700mg/kg | GLU, OGTT, p-PERK, p-IRE1↓ | in vivo | (Wang et al., 2007) |
|  | LX-2 cells | 25,300ug/ml | TGF-β1,HGF, MMP9, IL-10↓, MMP-2↑ | in vitro | (Li et al., 2008) |
| High-fat diets | C57BL / 6J mice | 8%(add into fat diets) | TC, AST, ALT, FBG, PINS↓, Shannon index, microflora diversity↑, the relative abundance of Firmicutes, Deferribacteres, synergistetes phyla, and the relative abundance of Bacteroidetes phylum reduced | in vivo | (Hong et al., 2020) |
| High-fat diets, 100nM FFAs | C57BL / 6J mice，HepG2 cells | 800mg/kg | Hepatic lobule structure improved, lipid accumulation deduced, BW, liver weight, GTT, ITT, FBG, LDL-C, TG, TC, ALT, AST, TNF-α, IL-6, IL-1β, LEPTIN, p-mTOR, p-4EP1, p-S6K1, Grp78↓, HDL-C, ADIPONECTIN, GLUT2, IRS-1, AMPK, ACC, AKT, GSK3β, PI3K, PEPCK, G6pase, Bcl-2, BAX, ATG3, ATG12, BECLIN-1, LC3-II/LC3-I↑ | in vitro, vivo | (Sun et al., 2019) |
| High-fat diets | GK rats | 500mg/kg | Lipid droplets deduced, BW, BG, TC, TG, LDL, TNF-α, miR-103a-3p, miR-181a-5p, miR-384-3p, GRP78, CHOP, p-JNK, caspase-12↓, miR-203a-3p, miR-106b-5p, GRP78↑ | in vivo | (Wei et al., 2018) |
| 2ml/kg 50% CCL4,i.p. | Wistar rats | 40mg/day | Necrosis, inflammation and hepatocyte ballooning improved, AST, ALT, GSH, MDA, CD68↓, TNF-α, IL-β1↓, the autophagy activation in KCs：ATG7, LC3II↓, T-AOC, SOD, GSH-Px, GSH↑ | in vivo | (Hamid et al., 2017) |
| 2ml/kg 50% CCL4,i.p. | Wistar rats | 40mg/day | Mild to moderate degree of hepatic necrosis and inflammation with partial infiltration of inflammatory cells and hepatocyte ballooning, liver weight/index, AST,ALT,ALP,LDH, α-SMA, Hyp, MDA, TNF-α, IL-6, COX-2, NF-κB, Collagen I, TGF-β1↓；BW, TP, T-AOC, GSH-Px, SOD, GPX1, SOD1, Nrf2, Bcl-2/BAX↑ | in vivo | (Hamid et al., 2017) |
| 100 mg/kg STZ, i.p. | NIH Swiss outbred mice | 200mg/kg | BW, FBG, PINS, IL-6, TNF-α, CCL2↓, OGTT↑ | in vivo | (Cui et al., 2016) |
| 5ml/kg 10% CCL4, s.c. | KM mice | 1g/kg | ALT, AST, MDA↓, SOD↑ | in vivo | (Pu et al., 2015) |
| High-fat diets | SD rats | 700mg/kg | the lipid droplet deposition deduced, BW, liver weight, PINS, glucose tolerance curves, BG, HOMA-IR, Fins，PGC1α↓, FGF21, PPARα, SIRT1↑ | in vivo | (Gu et al., 2015) |
| High-fat diets | C57BL / 6J, KKay mice | 700mg/kg | BW and hepacellular edema deduced, PINS, FBG, PBG, TG↓, IRS-1↑ | in vivo | (Ye et al., 2014) |
| High-fat diets | Golden Syrian hamsters | 0.25g/kg | Fractional cholesterol absorption rate, liver fatty degeneration deduced，cholesterol synthesis rates, bile acids increased, TC, TG, AST, ALT, LDL-C, HDL-C↓, LDLR, CYP7A1, HMG-CoA↑ | in vivo | (Cheng et al., 2011) |
| High-fat diets, 25mg/kg STZ, i.p. | SD rats，C2C12 cells | 700mg/kg；50,100,150,200,400μg/ml | Glycogen synthesis: hepatic glycogen content, glycogen particles increased, FBG, OGTT, HOMA-IR index, P-GS↓, ACC, GLUT4, p-AMPKα, AMPKα↑ | in vivo | (Zou et al., 2009) |
